# Transmission of Equine Influenza Virus to English Foxhounds

**DOI:** 10.3201/eid1403.070643

**Published:** 2008-03

**Authors:** Janet M. Daly, Anthony S. Blunden, Shona MacRae, Jodi Miller, Samantha J. Bowman, Jolanta Kolodziejek, Norbert Nowotny, Ken C. Smith

**Affiliations:** * Animal Health Trust, Kentford, Newmarket, Suffolk, UK; † University of Veterinary Medicine, Vienna, Austria

**Keywords:** Interspecies transmission, equine influenza A H3N8 virus, dogs, dispatch

## Abstract

We retrospectively demonstrated that an outbreak of severe respiratory disease in a pack of English foxhounds in the United Kingdom in September 2002 was caused by an equine influenza A virus (H3N8). We also demonstrated that canine respiratory tissue possesses the relevant receptors for infection with equine influenza virus.

Influenza A viruses are divided into subtypes according to the serologic reactivity of the surface glycoproteins hemagglutinin (H1–H16) and neuraminidase (N1–N9). Aquatic birds are regarded as the natural reservoir for influenza A viruses; a few mammalian hosts are infected by a limited number of virus subtypes. The first evidence of the H3N8 subtype, which currently circulates in horses, crossing species barriers was reported after an outbreak of respiratory disease among racing greyhounds in Florida in 2004. Isolation of virus from 1 case and detection of specific antibodies in other cases identified equine influenza virus as the cause of the outbreak (*1*). This information led us to reexamine an outbreak of severe respiratory disease that occurred in a pack of 92 English foxhounds in the United Kingdom in September 2002.

## The Study

The outbreak was signaled by a sudden onset of coughing. Some hounds became lethargic and weak; in some, these signs progressed to loss of consciousness. One hound died and 6 were euthanized. Postmortem examination of the hound that died (case 1) and 1 that was euthanized (case 2) showed subacute broncho-interstitial pneumonia; virus was suspected as the cause. When they were puppies (≈8 weeks of age), the hounds had been inoculated with commercially available vaccines against the major canine respiratory and enteric viruses. Postmortem tissue samples submitted to a canine infectious diseases laboratory were negative for known canine viral pathogens (e.g., canine herpesvirus, adenovirus, parainfluenza virus). The diagnosis as to the cause of the pneumonia, returned in 2002, was “unknown, suspected viral etiology.”

In January and March 2005, serum samples were obtained from the hounds affected by the respiratory disease outbreak in 2002 (pack 1). Serum samples were obtained from another 3 packs of foxhounds in the same region of the United Kingdom during December 2004 through February 2005. Samples were collected from 31–33 hounds (equivalent numbers of males and females) in each pack, ranging in age from 9 months to 9 years. The serum was screened for antibodies by using the single radial hemolysis assay (*2*). None of the samples contained antibodies to the strains that were included in the assay to control for nonspecific reactivity: equine H7N7 subtype strain A/equine/Prague/56 and the human influenza virus strain A/Puerto Rico/8/34 (H1N1). Antibodies to the H3N8 subtype strains A/equine/Newmarket/1/93 and A/equine/Newmarket/2/93 were, however, detected in 9 of the samples obtained during the first visit to pack 1 (Table). Of these, 8 were from hounds that had survived the outbreak in 2002; however, 1 was from a hound (no. 22) born after the outbreak in another part of the United Kingdom, which suggests that the 2002 outbreak might not have been the only incident of equine influenza to have infected hounds in the United Kingdom. Another 3 positive serum samples were obtained during a second visit to pack 1, and a repeat sample from hound no. 22 again had positive results. The specificity of the antibodies for equine influenza A (H3N8) strains was confirmed by hemagglutination inhibition assays that included human influenza (H3N2) strain A/Scotland/74 (data not shown).

**Table:** Antibody levels of English foxhounds involved in 2002 respiratory disease outbreak, United Kingdom*

Hound no.	Date sampled, 2005	Sex	Year born	Influenza virus strain	
				A/equine/Newmarket/1/93	A/equine/Newmarket/2/93
1	Jan 21	F	2002	<10	<10
2	Jan 21	M	2002	48	<10
2	Mar 9			46	<10
3	Jan 21	F	2001	<10	<10
4	Jan 21	F	2001	<10	<10
5	Jan 21	F	2002	47	21
5	Mar 9			64	36
6	Jan 21	M	2002	<10	<10
7	Jan 21	M	1999	<10	<10
8	Jan 21	M	2001	<10	<10
9	Jan 21	M	1998	43	<10
10	Jan 21	M	1999	76	54
11	Jan 21	M	1999	<10	<10
12	Jan 21	F	2002	55	28
13	Jan 21	M	1997	<10	<10
14	Jan 21	F	2003	<10	<10
15	Jan 21	M	2001	51	18
16	Jan 21	M	1999	<10	<10
17	Jan 21	F	2002	<10	<10
18	Jan 21	M	2002	13	11
19	Jan 21	M	1999	<10	<10
20	Jan 21	M	2001	<10	<10
20	Mar 9			<10	<10
21	Jan 21	F	2001	<10	<10
22	Jan 21	M	2003	52	25
22	Mar 9			71	40
23	Jan 21	M	2001	51	27
23	Mar 9			55	20
24	Jan 21	F	1999	<10	<10
24	Mar 9			<10	<10
25	Jan 21	F	2002	<10	<10
26	Jan 21	F	2000	<10	<10
26	Mar 9			<10	<10
27	Jan 21	F	1999	<10	<10
28	Jan 21	F	2000	<10	<10
29	Jan 21	F	2002	<10	<10
30	Jan 21	M	2002	<10	<10
30	Mar 9			<10	<10
31	Jan 21	F	1998	<10	<10
32	Jan 21	F	1999	<10	<10
33	Jan 21	M	2003	<10	<10
34	Mar 9	NK	NK	14	<10
35	Mar 9	NK	NK	<10	<10
36	Mar 9	NK	NK	99	54
37	Mar 9	NK	NK	<10	<10
38	Mar 9	NK	NK	<10	<10
39	Mar 9	NK	NK	<10	<10
40	Mar 9	NK	NK	83	33

*Measured by single radial hemolysis (mm^2^) in serum samples. NK, not known.

**Figure 1:**
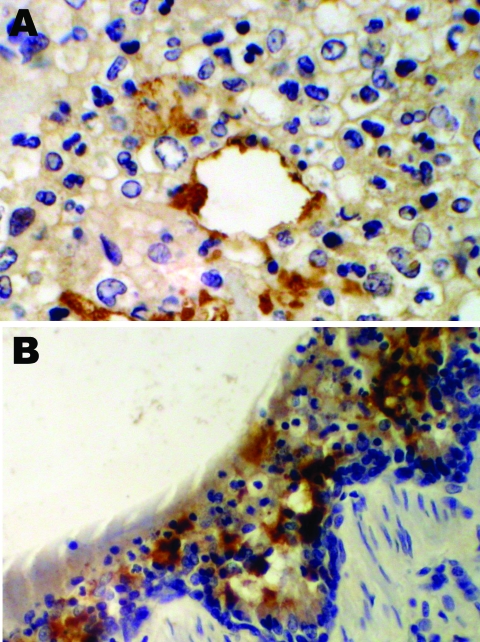
Immunohistochemical staining for equine influenza A virus (brown stain) in sections of respiratory tissue from English foxhounds involved in 2002 respiratory disease outbreak, United Kingdom. A) Case 1, showing focal staining of an apparently necrotic bronchiole in an area of pneumonia; magnification x100. B) Case 2, showing a large amount of staining throughout the epithelium and inflammatory cells present in the brush border; magnification x200; hematoxylin counterstain.

An immunohistochemical test to detect influenza A virus that used equine influenza–specific rabbit polyclonal antiserum was applied to formalin-fixed paraffin-embedded (FFPE) tissues from the 2 hounds that were examined postmortem in 2002 (*3*). Immunostaining of lung tissue showed positive staining in areas of pneumonic change; infected cells had the morphology of epithelial cells and macrophages (Figure 1). Immunostaining of visceral tissues (lung, liver, spleen, myocardium, intestine, pancreas, and oropharynx) was negative.

Deparaffinization of the FFPE lung tissue from the 2 hounds was performed as described previously (*4*) with a few modifications. RNA was extracted from the sample pellets obtained using the QIAamp viral RNA Mini Kit (QIAGEN, Hilden, Germany) according to the manufacturer’s instructions. Ten different primer pairs designed to amplify short (<250-bp) products from the matrix hemagluttinin and neuraminidase genes were used (details available from the authors on request). Reverse transcription–PCR (RT-PCR) was carried out by using the QIAGEN OneStep RT-PCR Kit. Only 1 primer pair (forward: 5′-AGGCAGGATAAGCATATACT-3′ and reverse: 5′-GTGCATCTGATCTCATTACA-3′, amplifying nucleotides 735–871 of the hemagglutinin gene) yielded an amplification product, which was purified by using PCR Kleen Spin Columns (Bio-Rad, Hercules, CA, USA) and sequenced by using the BigDye Terminator v1.1 cycle sequencing kit (Applied Biosystems, Foster City, CA, USA). A BLAST search with the 74-bp nt sequence obtained from this amplicon confirmed that the virus shared 100% identity with the equine influenza (H3N8) strains Newmarket/1/93 (*5*) and Newmarket/5/03 (*6*). This region contains 2 phylogenetically informative sites. Lysine at position 261 indicates that the virus belongs to the American lineage (*5*); this is supported by the presence of isoleucine at position 242 because all European lineage strains isolated since 1998 have valine at 242.

## Conclusions

**Figure 2:**
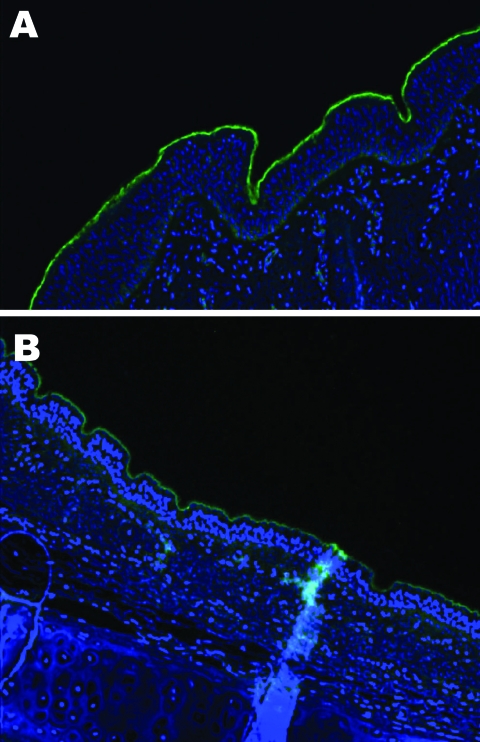
Lectin staining for α2,3 sialic acid linkages on A) equine trachea and B) canine trachea; magnification x200; cell nuclei counterstained with Hoechst 33342 solution.

An important factor in interspecies transmission is the ability of the hemagglutinin protein of the virus to bind to certain receptors on the host cells before the virus is internalized. Although all influenza A viruses recognize cell surface oligosaccharides with a terminal sialic acid, their receptor specificity varies; it is thought that species-specific differences in the distribution of linkages on respiratory epithelial cells influences the ability of influenza A viruses to transmit between species. Respiratory tract tissue samples were obtained within 2–4 hours of death from a horse and a greyhound, each euthanized for reasons other than this study, and rinsed extensively to remove surface mucous. The tissues were stained by immunofluorescence by using the lectins Sambucus nigra (SNA, specific for SAα2,6 galactose(Gal)/N-acetylgalactosaminide) and *Maackia amurensis* (MAA, specific for SAα2,3) as previously described (*7*). The MAA lectin bound strongly to the equine tracheal epithelium (Figure 2, panel A), which confirms the finding that the NeuAc2,3Gal linkage preferentially bound by equine influenza viruses is found on sialyloligosaccharides in the equine trachea (*7*). The MAA lectin also bound strongly to the canine respiratory epithelium (Figure 2, panel B) at all levels of the respiratory tract examined (distal, medial and proximal trachea; primary and secondary bronchi), which suggests that receptors with the required linkage for recognition by equine influenza virus are available on canine respiratory epithelial cells, although further subtle differences in receptor specificity may exist. The SNA lectin, specific for SAα2,6Gal, which did not bind to the equine tracheal epithelium, showed some binding to the canine epithelium (data not shown).

Because the hounds infected in 2002 were housed near horses, it is possible that the virus was transmitted from infected horses by the usual (aerosol) route. However, during the week before onset of clinical signs, the hounds had been fed the meat of 2 recently euthanized horses from independent sources. That viral antigen expression was confined to the lungs indicates a respiratory rather than oral route of infection. It is possible that eating respiratory tissue from an infected horse led to inhalation of sufficient virus particles to initiate a respiratory infection. Consumption of infected bird carcasses has been implicated in the transmission of highly pathogenic avian influenza virus of the H5N1 subtype to tigers and leopards (*8*) and a dog (*9*) and was demonstrated experimentally by feeding virus-infected chicks to domestic cats (*10*).

Although the mechanism remains unclear, we have demonstrated transmission of equine influenza virus to dogs in the United Kingdom, independent of that in the United States. We have also shown that canine respiratory tissue displays the relevant receptors for infection with equine influenza virus.
